# Analysis of the Genome Sequence and Prediction of B-Cell Epitopes of the Envelope Protein of Middle East Respiratory Syndrome-Coronavirus

**DOI:** 10.1109/TCBB.2017.2702588

**Published:** 2017-05-29

**Authors:** Qian Xie, Xiaoyan He, Fangji Yang, Xuling Liu, Ying Li, Yujing Liu, ZhengMeng Yang, Jianhai Yu, Bao Zhang, Wei Zhao

**Affiliations:** 1 Guangzhou Key Laboratory of Drug Research for Emerging Virus Prevention and TreatmentGuangdong Provincial Key Laboratory of Tropical Disease ResearchSchool of Public Health Southern Medical University No. 1023 Shatai RoadGuangzhou510515P.R. China; 2 Nanfang HospitalSouthern Medical UniversityNo. 1023, Shatai RoadGuangzhou510515P.R. China; 3 Key Laboratory of Liver Disease of Guangdong ProvinceDepartment of Infectious DiseasesThe Third Affiliated Hospital Sun Yat-sen UniversityGuangzhou510515P.R. China

**Keywords:** MERS-CoV, bioinformatics, linear B-cell epitope

## Abstract

The outbreak of Middle East respiratory syndrome-coronavirus (MERS-CoV) in South Korea in April 2015 led to 186 infections and 37 deaths by the end of October 2015. MERS-CoV was isolated from the imported patient in China. The envelope (E) protein, a small structural protein of MERS-CoV, plays an important role in host recognition and infection. To identify the conserved epitopes of the E protein, sequence analysis was performed by comparing the E proteins from 42 MERS-CoV strains that triggered severe pandemics and infected humans in the past. To predict the potential B cell epitopes of E protein, three most effective online epitope prediction programs, the ABCpred, Bepipred, and Protean programs from the LaserGene software were used. All the nucleotides and amino acids sequences were obtained from the NCBI Database. One potential epitope with a suitable length (amino acids 58–82) was confirmed and predicted to be highly antigenic. This epitope had scores of >0.80 in ABCpred and level 0.35 in Bepipred programs. Due to the lack of X-ray crystal structure of the E protein in the PDB database, the simulated 3D structure of the E protein were also predicted using PHYRE 2 and Pymol programs. In conclusion, using bioinformatics methods, we analyzed the genome sequence of MERS-CoV and identified a potential B-cell epitope of the E protein, which might significantly improve our current MERS vaccine development strategies.

## 1 Introduction

Middle East respiratory syndrome (MERS, so the early once called it a kind of virus like SARS) is a newly described disease in humans and was first reported after the identification of a novel beta coronavirus (MERS-CoV) from a patient who died of a severe respiratory illness in Saudi Arabia in September 2012 [1]. Coronavirus is a canonical and ancient virus system that can be divided into four categories based on their genome sequence: *Alphacoronavirus*, *Betacoronavirus*, *Gammacoronavirus* , and *Deltacoronavirus* coronavirus. Since it was discovered coronavirus was considered to be relatively harmless to humans until the outbreaks of SARS and MERS in 2003 and 2012, respectively. MERS-CoV is a new type of coronavirus identified after the discovery of SARS-CoV, belongs to the *Beta* coronavirus lineage C [2], which causes severe acute respiratory disease with a high fatality rate. Moreover, the virus rapidly spread from the Middle East to many other countries including South Korea in 2015, demonstrating a global epidemic trend. However, no effective antiviral drug or vaccine has been developed to treat MERS-CoV.

Coronavirus is a class of enveloped RNA virus with a 27–3l kb long single-stranded positive-sense genome. The genome includes two large replicase open reading frames, ORF1a and ORF1b, encoding two viral replicase polyproteins. The region downstream of ORF1 contains at least 10 small ORFs, encoding the spike protein (S), small envelope protein (E), membrane protein (M), nucleocapsid protein (N) and the assumed nonstructural proteins [3]. Among these proteins, the E protein is a relatively smaller but massively expressed virus envelope protein and plays an important role in virus membrane packaging [4]. MERS-CoV is a new type coronavirus with a 30.1 kb long genome. Similar to the other coronaviruses, the E protein is a small structural protein in MERS-CoV which was identified as the structural component of the virus. Previous studies suggest that the E proteins are scattered on the surface of the virus envelope and play various important roles in regulating the viral life cycles in some coronaviruses [3], [5], [6], [7]. Understanding the MERS-CoV E protein structure and function could possibly find therapeutic targets to prevent and control the coronaviruses related diseases.

Using bioinformatics methods, we analyzed the MERS CoV E protein's sequence and its secondary and 3D structures. E protein was found to be stable, hydrophobic and highly conserved among different coronavirus stains. Various secondary structures were identified in E protein and its 3D structure was predicted. Finally one potential B-cell linear epitope amino acids position 58-82 was identified at C-terminal of E protein. Thus our study suggests that E protein could possibly be a good candidate for B cell-line epitopes in preparing monoclonal antibodies, vaccines and anti-viral inhibitors against MERS-CoV infection in the future.

## 2 Methods

### 2.1 Genome Sequences Comparison Analysis

**Fig. 1.**
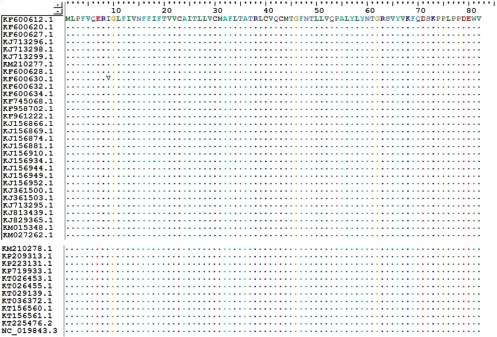
Sequence alignment of 42 strains coronavirus E protein.

The sequences of MERS-CoV E protein and other coronavirus E proteins were also compared using the Clustal W multiple sequence alignment program (Bioedit 7). The nucleotide and amino acid sequences of the E proteins from 42 MERS-CoV strains (Fig. 1) were aligned using the Clustal W multiple sequence alignment program (Bioedit 7). All sequences are available at the NCBI database (http://www.ncbi.nlm.nih.gov/nucleotide/ ).

### 2.2 Analysis of Protein Primary Structure and Physical Properties of the MERS-CoV E Protein

The complete sequence of the MERS-CoV E protein contains 82 amino acids(GeneBank ID: AGV08384.1) was obtained from the NCBI database. General physical properties of the E protein, including the theoretical pI value, amino acid composition and molecular weight, were analyzed using the online ProtParam tool (http://web.expasy.org/protparam/ ).

### 2.3 Prediction of Secondary Structure and B Cell Linear Epitope

The secondary structure and linear B-cell epitopes of the E protein were predicted by ABCpred, Bepipred, and Protean programs in LaserGene software. Selected sequences were downloaded from NCBI database and analyzed by each program. ABCpred uses a recurrent method which is based on a neural network algorithm [8] (http://www.imtech.res.in/raghava/abcpred/ABC_submission.html). The lengths of amino acids were set to 12-mer, 14-mer, 16-mer, 18-mer, and 20-mer and the scoring threshold was set to 0.8. Since there is no evidence showing that 20 amino acids is the optimal length for B-cell epitopes, a length of 15–20 amino acids is generally considered. Optimal length is determined by the overall score, higher score indicates a better chance that a sequence is an antigen epitope. Bepipred was developed by Larsen et al. [9] ( http://www.cbs.dtu.dk/services/BepiPred/) and it implements a hidden Markov model and a propensity scale method as described by Parker et al. [10]. A threshold of 0.350 was used. For this threshold the sensitivity is 0.49 and the specificity is 0.75. The Protean program in LaserGene software uses the Garnjer-Robson, Chou-Fasman and Karplus-Schulz methods to predict the protein's secondary structure. The parameters of each residue were determined by Pα, Pβ, PT, and PC values [11]. According to the Kyte-Doolittle standard for hydrophilic amino acids, potential B cell linear epitopes were usually the hydrophilic regions on the protein surface (as described by Emini). Karplus-Schulz method was used to identify the flexible regions of the E protein. Jameson-Wolf method was used to predict the antigenic index. The significance of the parameters has been described previously [12], [13], [14]. All the resulting sequences were collected and aligned using Clustal W multiple sequence alignment programs. Overlapped regions were considered as the potential epitopes.

### 2.4 Prediction of 3D Structure

PHYRE 2 server was used to model multiple domains of the MERS-CoV E protein with high confidence based on the high scoring template [15]. The predicted 3D structures were delivered through emails and downloaded as PDB files, which were further visualized and modeled using the molecular modeling tool PyMOL (Version 1.7.4 Schrödinger, LLC) [16]. All the final 3D structures were generated as ball-and-stick models with distinct colors using PyMOL as previously described [17].

## 3 Results

### 3.1 MERS-CoV E Protein is Highly Conserved Among Different Coronavirus Stains

MERS-CoV strain was compared with the other 41 coronavirus stains including SARS coronavirus (SARS-CoV, NP_828854), human coronavirus (HCoV 229E, NP_073554.1), canine coronavirus (CcoV, BAA02412.1), cat coronavirus (FCoV, CAA74228. 1), bovine coronavirus (BCoV, AAL40404. 1), etc (Table 1). Whole genome sequences among those host specific coronaviruses shared less than 30 percent similarities. In contrast, further sequence alignment analysis showed that the E protein sequences were nearly identical among the 42 MERS-CoV strains except one single point mutation from isoleucine to valine at amino acid position 9 (I9V) ( Fig. 1). Moreover, since isoleucine and valine residues both are hydrophobic, the I9V point mutation may not alter the antigenic behavior of the E protein. Together these results suggest that that E protein of MERS-CoV is highly conserved among different virus strains.

**TABLE 1:** Comparison with Other Coronavirus

Virus	Protein ID	Number of amino acids	Blast with MERS-CoV
SARS CoV	NP_828854	76	37.80%
HCoV	NP_073554.1	77	22.62%
CCoV	BAA02412.1	82	18.60%
FCoV	CAA74228.1	82	22.99%
BcoV	AAL40404.1	84	27.38%
RTCoV	AAF97741.1	88	25.00%
MHV	NP_068673	83	23.81%
IBV	AAO33465.1	103	15.60%

### 3.2 The E Protein of MERS-CoV is Stable and Hydrophobic

ProtParam analysis of MERS-CoV E protein suggests that its theoretical pI value is 7.64 and the molecular weight is 9354.2 daltons. The computed instability index (II) is 33.00, suggesting that MERS-CoV E protein is classified as a stable protein (Protein with II values <40 is considered stable). Moreover, the grand average for hydropathicity (GRAVY) value is 0.795, indicating that E protein is hydrophobic (Protein with positive GRAVY value is hydrophobic while it is considered hydrophilic with negative GRAVY value).

### 3.3 Identification of Potential B-Cell Linear Epitopes

Various lengths of the polypeptides were set for epitope prediction by ABCpred program, as described in methods. Higher scores reflected a higher probability of being identified as a B-cell epitope. Eight linear B-cell epitopes in the MERS-CoV E protein showed scores above the threshold value of 0.8. Scores beyond 0.8 mean to be positive, as shown in Table 2. Meanwhile, one epitope peptide was identified by Bepipred 1.0 server, as shown in Table 3. We used 0.35 as the threshold. Scores beyond 0.35 account for potential epitote, and a higher score indicates a higer probability of the existing epitope. Although the resulting epitope peptides from ABCpred and Bepipred were not totally identical, the overlapping regions were considered as the B-cell line epitope areas. As shown in Table 3, epitopes 2, 5, and 7 were identified as the potential B-Cell line epitopes.

**TABLE 2:** Potential Epitopes (by ABCpred)

Rank	Sequence	Position	Length	ABCpred score
1	VVCAITLLVCMAFLTA	21–36	16	0.89
2	TGRSVYVKFQDSKP	61–74	14	0.88
3	AFLTATRLCVQCMTGFNT	32–49	18	0.84
4	LFIVNFFIFTVVCAITLLVC	11–28	20	0.82
5	RSVYVKFQDSKPPLPP	63–78	16	0.81
6	LCVQCMTGFNTLLVQP	39–54	16	0.81
7	TGRSVYVKFQDSKPPLPP	61–78	18	0.81
8	AFLTATRLCVQCMT	32–45	14	0.80

**TABLE 3:** Details of Bepipred Prediction

Rank	Sequence	Position location	Length	Mean residue score
1	QDSKPPLPPDEWV	70–82	13	1.687

**Fig. 2.**
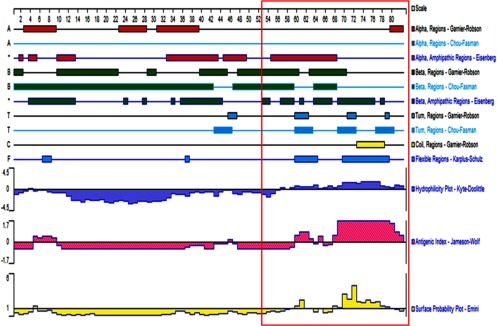
Secondary structure and B cell line epitope presented by Protean program. Secondary structure was predicted using Garneier-Robson, Chou-Fasman, and Eisenberg methods. Red, green, and light blue regions represent alpha helix, beta lamella, and T-corner, respectively. Flexible region, hydrophilicity plot, antigen index, and surface possibility are highlighted with blue, pink and yellow, respectively. The area of the antigen epitope is marked by a red-frame box.

Structural analysis following the Kyte-Doolittle standard suggests that a large hydrophilic area was presented in the amino acids (aa) 58–82 region at the C-terminal of E protein. Further study of Jameson-Wolf antigenicity index showed that the highest indices were from aa 3–10, aa 58–64, and aa 68–82 regions. Moreover, analysis using Emini methods indicated that aa 67–82 epitope was possibly presented on the protein surface ( Fig. 2). Taken together, since the overlapped regions were considered as the potential epitopes, AA 58–82, with its amino acid sequence of LYNTGRSVYVKFQDSKPPLPPDEWV, was identified as a potential B cell linear epitope of the E protein.

### 3.4 Secondary Structure of the E Protein of MERS-CoV

The secondary structures obtained using the Garneier-Robson, Chou-Fasman and Eisenberg methods were not consistent, as shown in Fig. 2. Various secondary structures (i.e., alpha helix, beta lamella, T-corner and coils) were analyzed using the Garneier-Robson method, the Chou-Fasman method and the Eisenberg method. Further study following the Karplus-Schulz method suggests that there are three flexible areas in the E protein, i.e., aa 6–12, aa 58–65 and aa 69–80 (Fig. 2). Those areas are predicted to be exposed on the surface of MERS-CoV, suggesting their roles as potential antigen epitopes, supporting our previous identification of aa 58–82 as a potential B cell linear epitope of the E protein.

### 3.5 Prediction of the 3D Structure of the E Protein of MERS-CoV

**Fig. 3.**
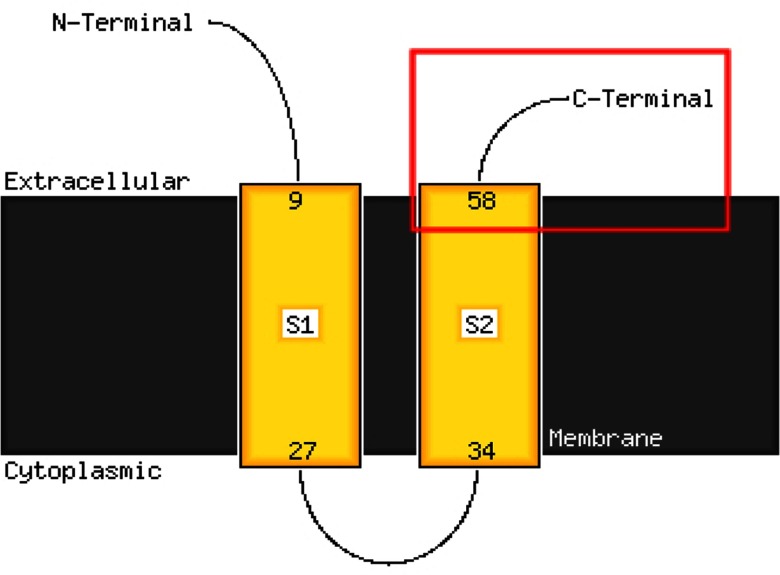
Predicted transmembrane helix regions of the E protein by PHYRE 2. Cell membrane is presented as the gray shaded area. S1 and S2 are transmembrane domains of the E protein. Predicted B-cell line epitope was presented in the red-frame box.

**Fig. 4.**
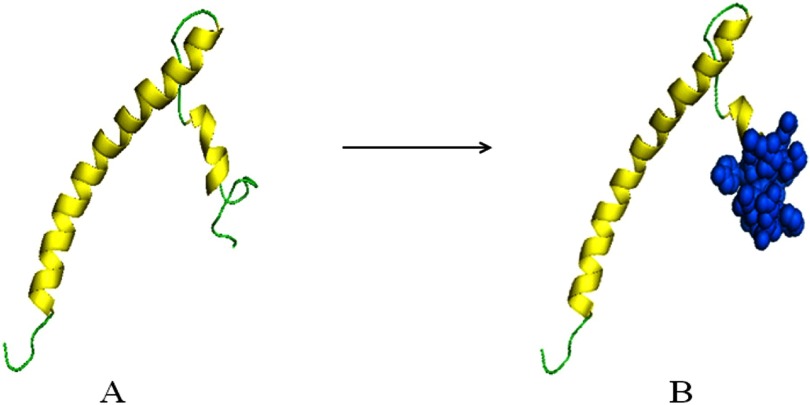
Predicted protein modeling of the E protein. Helix is highlighted in yellow and loops is presented in green in model A. Amino acids 58–82 region is shown as marine blue spheres in model B.

Since the 3D structure for the MERS-CoV E protein is unavailable in the PDB Molecule Database, PHYRE 2 server was used to predict and model multiple domains of E protein with high confidence based on the high scoring template. Predicted 3D structure of the E protein generated by PHYRE 2 server presented the Chain A of the E protein at the highest confidence level of 99.6 percent in the PDB Molecule Database. Further study using PHYRE 2 online server indicated that the N-terminal aa 1–9 and C-terminal aa 58–82 regions could possibly be located on the cell surface with two transmembrane domains S1 and S2 linked by a re-entrant helix, while the transmembrane helices were predicted to adopt the topology, as shown in Fig. 3. Final structure of the MERS-CoV E protein was generated by PyMOL, as shown in Fig. 4. The alpha helix was highlighted with yellow color while the loop was in green in model A, aa 58–82 regions was presented as marine blue spheres in model B.

## 4 Discussion

The full length natural antigens, particularly the hydrophobic membrane proteins, are hardly overexpressed and purified from prokaryotic cells with antigenicity. Previous studies suggest that protein antigenicity is generally determined by its specified epitopes instead of the full length sequence [18] . To identify the antigen epitopes, bioinformatics methods are used to predict their sequences. Predicted epitopes are further synthesized *in vitro* and validated with experiments. Dozens of B cell antigen epitopes have been discovered following various algorithms, offering us multiple reliable means of predicting the antigen's hydrophilic regions, accessibility, flexibility and antigenic solubility [19] . The ABCpred algorithm, which was used to predict the linear B-cell epitopes of MERS-CoV E protein in our study, has been previously shown to successfully predict epitopes with 65.93 percent accuracy ( http://www.imtech.res.in/raghava/abcpred/) [20]. Thus bioinformatics studies could provide reliable guidance in selecting specific immunogenic epitopes, which will be significant for vaccine design, epitope mapping and antibody studies. Further combination of various bioinformatics prediction methods could significantly increase the prediction accuracy.

Recent outbreak of MERS-CoV in South Korea was the largest one outside of Saudi Arabia in the world [21]. Because the mortality rate of the MERS-CoV is as high as 40 percent, it is considered as one of the most critical emerging pathogens threatening human health. Previous study showed that a recombinant SL-CoV containing a very small fragment of the SARS-CoV S gene was able to infect and cause disease in mice, highlighting its potential for pathogenicity in humans [22]. However, characterization of the immunogenic determinants for the MERS-CoV protein remains unresolved. Here in our study we found that the gene sequences of MERS-CoV were moderately conserved among different coronavirus strains although Cotten et al. studied MERS-CoV gene diversity and found numerous variations among different strains [23]. Nevertheless, at least two distinct lineages, including circulating and transmission patterns in the epidemic, are consistent in both human-to-human transmission and sporadic zoonotic events. The E protein, which is essential for virus packaging, is a moderately expressed small transmembrane protein and presented on the surface of virus envelope as well as the infected cells. Our further comparison studies showed that the E protein is highly conserved among different virus strains isolated from different species (e.g., Camelus dromedarius, Vespertilio superans and Homo sapiens) at different times and locations near the endemic areas [24]. Since sequence alignment revealed that the E protein from the 42 strains were nearly identical except a point mutation from isoleucine to valine. And Durai P [25] had stated that MERS-CoV E protein has striking similarities to SARS-CoV E protein, which has a resolved NMR structure. Therefore E protein was chosen as a potential antigenic target for the humoral immune responses, which might be significant for developing better diagnostic and research reagents in the future.

To further identify the potential antigen epitope regions of the E protein, ABCpred, Bepipred and the protean package in LaserGene software were used to predict the E protein secondary structure as well as B cell epitopes by overlapping the sequences generated from the three methods. Based on our results, aa 58–82 was identified as a potential antigen epitope region. Further online prediction and sequence comparison analysis revealed that this region was highly antigenicity and low variation region, supporting our prediction of AA 58–82 as an antigen epitope region. Since E protein structure is not available in the PDB molecular database, we used the PHYRE 2 server and PyMOL software to predict the 3D structure of E protein. The predicted structure was consistent with previous secondary structure prediction. AA 58–82 of the E protein was predicted to be a good candidate as the B cell epitope peptide area. Overall, using bioinformatics methods, we have successfully identified a potential B cell line epitope of MERS-CoV E protein, while experimental and clinical evidence are necessary to validate our studies. Future studies could also relate to other proteins in MERS-CoV and other coronavirus strains. Since currently there is no effective treatment or preventive vaccine available targeting MERS-CoV, the fusion B-cell epitopes of the E protein identified in this study might be the potential targets to design effective MERS-CoV vaccines and facilitate the development of rapid diagnostic methods in the future.
